# The PREvention Program for Alzheimer’s RElated Delirium (PREPARED) cluster randomized trial: a study protocol

**DOI:** 10.1186/s12877-021-02558-3

**Published:** 2021-11-16

**Authors:** Machelle Wilchesky, Stephanie A. Ballard, Philippe Voyer, Jane McCusker, Ovidiu Lungu, Nathalie Champoux, T. T. Minh Vu, Martin G. Cole, Johanne Monette, Antonio Ciampi, Eric Belzile, Pierre-Hugues Carmichael, Ted McConnell

**Affiliations:** 1 Centre for Research in Aging, Donald Berman Maimonides Geriatric Centre, 5795 Ave Caldwell, Montreal, QC H4W 1W3 Canada; 2 Department of Family Medicine, McGill University, 5858 Côte-des-Neiges Road, Montreal, QC H3S 1Z1 Canada; 3 Division of Geriatric Medicine, McGill University, Jewish General Hospital, 3755 Cote St. Catherine Road, Room E-0012, Montreal, QC H3T 1E2 Canada; 4 Centre for Clinical Epidemiology, Lady Davis Institute, Jewish General Hospital, 3755 Cote St. Catherine Road, Montreal, QC H3T 1E2 Canada; 5 Faculty of Nursing Sciences, Laval University, Pavillon Ferdinand-Vandry, 1050 avenue de la Médecine, local 3645, Québec, QC G1V 0A6 Canada; 6 Centre d’excellence sur le vieillissement de Québec, Centre intégré universitaire de santé et de services sociaux de la Capitale-Nationale, 1050 chemin Sainte-Foy, L2-30, Quebec City, QC G1S 4L8 Canada; 7 Department of Epidemiology, Biostatistics and Occupational Health, McGill University, 1020 Pine Ave W, Montreal, QC H3A 1A2 Canada; 8 St. Mary’s Research Centre, 3830 Avenue Lacombe, Hayes Pavilion, suite 4720, Montreal, QC H3T 1M5 Canada; 9 Functional Neuroimaging Unit, Centre de recherche de l’Institut universitaire de gériatrie de Montréal, 4565 Queen Mary Rd, Montreal, QC H3W 1W5 Canada; 10 Department of Psychiatry, Université de Montréal, Pavillon Roger-Gaudry, Faculté de Medicine, C.P. 6128, succursale Centre-ville, Montreal, QC H3C 3J7 Canada; 11 Faculty of Medicine, Université de Montréal, 2900 Boulevard Edouard-Montpetit, Montreal, QC H3T 1J4 Canada; 12 Centre de recherche du CHUM, 91000, rue Saint-Denis, Montréal, QC H2X 0A9 Canada; 13 Department of Psychiatry, McGill University, Ludmer Research & Training Building, 1033 Pine Avenue West, Montreal, QC H3A 1A Canada; 14 Division of General Internal Medicine, McGill University, 1001 Decarie Boulevard, Montreal, QC H4A 3J1 Canada

**Keywords:** Delirium, Delirium superimposed on dementia, Modifiable risk factors, Multicomponent intervention, Nursing, Long-term care

## Abstract

**Background:**

Delirium is a significant cause of morbidity and mortality among older people admitted to both acute and long-term care facilities (LTCFs). Multicomponent interventions have been shown to reduce delirium incidence in the acute care setting (30–73%) by acting on modifiable risk factors. Little work, however, has focused on using this approach to reduce delirium incidence in LTCFs.

**Methods:**

The objective is to assess the effectiveness of the multicomponent PREPARED Trial intervention in reducing the following primary outcomes: incidence, severity, duration, and frequency of delirium episodes in cognitively impaired residents. This 4-year, parallel-design, cluster randomized study will involve nursing staff and residents in 45–50 LTCFs in Montreal, Canada. Participating public and private LTCFs (clusters) that provide 24-h nursing care will be assigned to either the PREPARED Trial intervention or the control (usual care) arm of the study using a covariate constrained randomization procedure. Approximately 400–600 LTC residents aged 65 and older with dementia and/or cognitive impairment will be enrolled in the study and followed for 18 weeks. Residents must be at risk of delirium, delirium-free at baseline and have resided at the facility for at least 2 weeks. Residents who are unable to communicate verbally, have a history of specific psychiatric conditions, or are receiving end-of-life care will be excluded. The PREPARED Trial intervention consists of four main components: a decision tree, an instruction manual, a training package, and a toolkit. Primary study outcomes will be assessed weekly. Functional autonomy and cognitive levels will be assessed at the beginning and end of follow-up, while information pertaining to modifiable delirium risk factors, medical consultations, and facility transfers will be collected retrospectively for the duration of the follow-up period. Primary outcomes will be reported at the level of intervention assignment. All researchers analyzing the data will be blinded to group allocation.

**Discussion:**

This large-scale intervention study will contribute significantly to the development of evidence-based clinical guidelines for delirium prevention in this frail elderly population, as it will be the first to evaluate the efficacy of a multicomponent delirium prevention program translated into LTC clinical practice on a large scale.

**Trial registration:**

NCT03718156, ClinicalTrials.gov.

## Background

Delirium is a severe neuropsychiatric syndrome with acute onset and a fluctuating course that is characterized by disturbances in cognition, consciousness and attention [1]. It is a significant cause of morbidity and mortality, and is highly prevalent among older individuals across healthcare settings (14–56% reported in acute care [2], 58–75.6% in intensive care units [3], and 1.4–70.3% in long-term care facilities (LTCFs)) [4]. Dementia is an important risk factor for developing delirium among elderly patients [5–7]: the risk of developing delirium is six-times greater among older individuals with dementia when compared to those without the disease [5], and prevalence rates of delirium superimposed on dementia range from 22 to 89% among populations aged 65 and older [8]. This is of particular concern in LTCFs, where dementia is common among the resident population [9]. Residents in LTCFs are also at an increased risk of developing delirium as a function of their frailty, cognitive impairment, and multiple medical comorbidities [10, 11]. Indeed, more than 40% of LTCF residents experience at least one delirium episode over the course of their stay [12], leading to an increased number of falls, accelerated functional decline, increased mortality, and higher healthcare costs [1]. Despite these consequences, delirium recognition is known to be poor. Up to two-thirds of delirium cases are missed by physicians and nurses due to its fluctuant and variable nature [13], ,and it has been reported that 87% of delirium episodes that are superimposed on dementia go undetected in LTCFs [14].

Delirium prevention is essential [15, 16], as the risk of failure to return to baseline among survivors is high, even after adjustment for factors such as age and pre-morbid function [17]. Previous studies have found that 30–40% of delirium cases are preventable with the use of delirium protocols [11], and one American study estimates potential healthcare savings of up to 16 billion dollars annually [18]. Several multicomponent delirium prevention programs (MDPP) have successfully reduced delirium incidence in the acute care setting (30–73% reduction) [1, 18–22]. Independent of type of acute-care ward or unit and level of cognitive decline, a 2015 systematic review of randomized trials of multicomponent interventions reported a relative reduction of 27% (RR 0.73, 95%CI: 0.63–0.85) in delirium incidence [1]. Similarly, a systematic review that analyzed non-randomized studies reported an overall reduction of 63% (RR 0.37, 95% CI: 0.27–0.53) [1]. Such interventions have also been associated with an overall reduction in hospital stay duration and mortality rate [23]. Although the wide range of estimates make it difficult to ascertain the degree to which delirium incidence can be reduced, it is clear that these interventions do have an impact in the acute care setting. The extent to which these findings can be extrapolated to the LTC setting, however, is unknown. For example, the studies listed in the above-mentioned acute care systematic reviews included patients who are younger than LTC residents (average age of 79 in acute care versus 85 in LTC) [1, 18], and who have shorter lengths of stay (average 5–38 days in acute care versus 2.5 years for LTC) [1, 18]. The prevalence of dementia is also different in acute care versus LTC (but this information is not available for the above-mentioned studies).

According to both a 2014 Cochrane Database Systematic Review and the National Institute for Health and Clinical Excellence, research to assess the effectiveness of an MDPP in LTC is urgently needed [24, 25]. Despite this call to action and the increased risk of developing delirium in the LTC population, no multicomponent intervention based on modifiable risk factors had been evaluated and translated into LTC clinical practice to date at the time that we conceived the present trial [19]. However, in 2020, Boockvar et al. published a trial measuring the efficacy of a different multicomponent intervention targeting delirium risk factors (specifically, cognitive impairment, immobility, dehydration, and malnutrition) in LTC, the Hospital Elder Life Program (HELP-LTC) [26]. The HELP-LTC targeted a limited sample size (*n* = 219), and the intervention did not demonstrate its intended effect: no significant differences were found in delirium or delirium severity between the intervention and usual care groups [26]. A multitude of factors are listed by the authors to explain this null finding, including baseline differences in cognitive function between groups, greater than expected improvements on delirium severity and cognition, and novel adaptations of the intervention [26].

In contrast, an integrated knowledge translation strategy [27] was used to develop the PREvention Program for Alzheimer’s RElated Delirium (PREPARED Trial) intervention for use in a LTC setting. Key stakeholders who were familiar with the LTC context were identified early in the research planning process and consulted throughout all subsequent research phases. To address the fact that interventions that are effective in clinical trials may underperform in a real world setting [28], a participatory approach was then used to examine and test the feasibility and acceptability of the PREPARED Trial intervention in two LTC facilities in three 5-week implementation cycles [29]. A thorough evaluation of the effectiveness of the PREPARED Trial intervention in preventing delirium among at-risk LTC residents is now required.

## Study objectives

The primary objective of this study is to compare the efficacy of the PREPARED Trial multicomponent intervention to that of usual care on delirium. Specifically, we aim to assess the effect of the PREPARED Trial multicomponent intervention on the *incidence* of delirium, *severity* of delirium episodes, *duration* of delirium episodes, and *number* of delirium episodes among at-risk, cognitively impaired residents in LTCFs. Our secondary objectives aim to: compare the efficacy of the PREPARED Trial intervention to that of usual care on the *incidence of falls* among cognitively impaired LTC residents; estimate the *association between medication use and delirium incidence* in LTCFs; estimate if there is an effect modification by motor subtype of delirium or by dementia subtype; and measure the prevalence of delirium in participating LTCFs. Finally, we will compare the effect of the multicomponent PREPARED Trial intervention on other health outcomes, including changes in: functional autonomy or social engagement, cognitive functioning, the number of transfers to acute care, the number of consultations with healthcare providers, and mortality rates. All objectives will be reported at the level of intervention assignment.

## Methods/design

### Study design

The PREPARED Trial is a 4-year cluster randomized trial. The PREPARED Trial intervention will be implemented at the LTCF level. To reduce potential contamination between intervention and control study arms by nursing staff members who may provide replacement hours of employment at different units/floors within their LTCF, a cluster design was adopted such that participating LTCFs (clusters) will be assigned to either the PREPARED Trial intervention or the control (usual care) arm of the study. Nursing staff and LTC residents meeting trial inclusion criteria from all arms will be enrolled in the study. Residents will be followed for an 18-week period or until death or transfer to another facility.

**Fig. 1:**
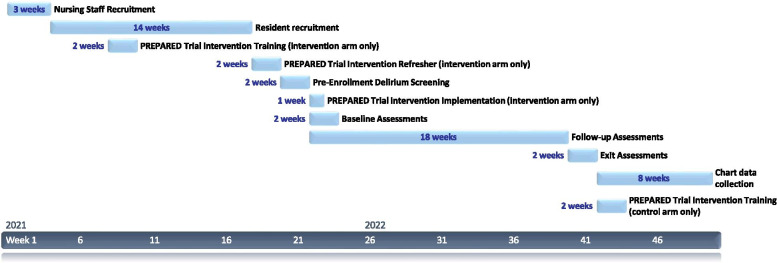
Timeline of Research Activities per Trial LTC Facility

A cluster randomized trial parallel design, employing a covariate constrained randomization (CCR) procedure [30, 31], will be used to allocate LTCFs, rather than individual residents, to either the intervention or control study arm. Cluster randomized trials typically use clusters as the main unit of analysis, given that the intervention is conducted at the cluster level (in the current study, the LTCFs are the clusters). However, given that the number of clusters is usually much smaller than the total number of participants, a simple cluster randomization or a procedure based on stratification may yield study arms that are imbalanced and differ on key baseline cluster characteristics. A CCR approach will be used to address this issue, as it specifically aims to balance the distribution of important cluster characteristics or covariates across study arms [30–32]. In addition, given the need to assess each resident weekly for a follow-up period of 18 weeks and the logistical limitations (large geographical coverage, 45–50 LTCFs included, and limited work force) involved in such a trial, we will employ a sequential approach whereby sets of clusters (LTCFs) will be randomized into the two study arms in consecutive trial ‘cycles’ lasting 20 weeks each, including the two-week screening period before enrolment (see Fig. 1 for a breakdown of research activities within a study cycle).

This 18-week period was selected using data from one of the precursor delirium studies conducted in this population, which found that 97% of all incident episodes of delirium among cognitively impaired residents occurred within the first 18-weeks of follow-up [33]. Research staff will visit each facility to enroll eligible residents and to conduct follow-up assessments. Data will also be abstracted from resident charts and existing LTCF databases.

### Participants

Participants for this study will include LTC residents (who will be assessed for delirium, cognition, functional autonomy, and other clinical/demographic variables) and nursing staff members (who will provide data pertaining to the resident population and/or implement the intervention). No monetary incentives will be provided to remunerate participation. Participating LTCFs will include public and semi-private residential LTCFs in and around Montreal, Quebec that provide residence to elderly individuals requiring specialized care per day, including assistance with one or more basic activities of daily living beyond that which can be provided in a community setting [34, 35]. Institutions will only be eligible if they provide 24-h of supervised nursing care (i.e. an RN is on site 24 h per day), including professional health services and personal care.

All Registered Nurses (RNs), Licensed Practical Nurses (LPNs) and personal support workers (PSWs) working during the day shift will be eligible to participate if they: 1) are working at least 3 days/week on the same nursing unit (i.e. are not ‘floating’ between units); and 2) have worked at the LTCF for at least 1 month prior to the follow-up start date at their site.

**Fig. 2:**
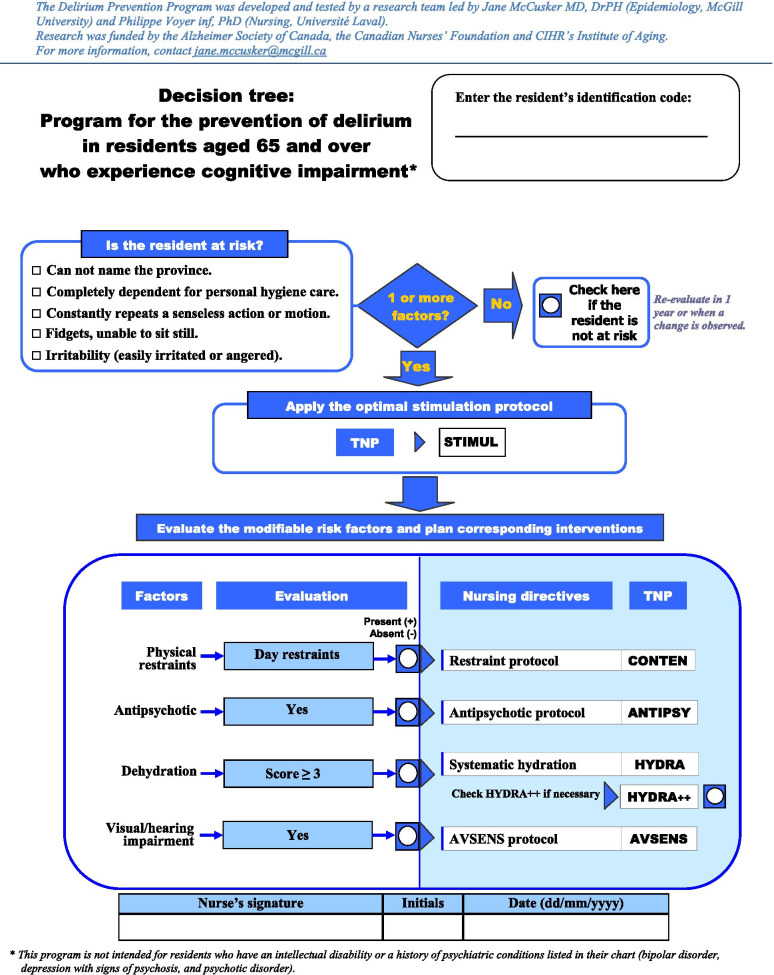
Delirium Risk Screening Tool and Decision Tree

Residents will be enrolled based on a series of inclusion/exclusion eligibility criteria. In order to be included, residents must: be 65 years or older; have dementia and/or cognitive impairment, as determined by discussions with the nursing staff and chart abstraction; have a minimum length of stay in the LTC facility of at least 2 weeks prior to the start of the baseline assessments; be at risk of delirium, as indicated by a score of 1 or higher on a validated 5-item delirium risk screening tool [36] (Fig. 2); and be delirium-free at baseline, as assessed by the Confusion Assessment Method (CAM) [37, 38], the Delirium Index (DI) [39], and a brief chart review over a period of two consecutive weeks immediately preceding the start of the follow-up period. Residents will be excluded if they are: unable to communicate verbally (as determined by either the nursing staff or two consecutive 0-score administrations of the composite cognitive interview, which is part of the CAM procedure); unable to communicate verbally in English or French; have a history of specific psychiatric conditions (bipolar disorder, depression with signs of psychosis, and psychotic disorders) or intellectual disability [40, 41]; or are being provided with end-of-life or palliative care.

### Sample size calculation

Based on sample size calculations, we plan to recruit between 400 and 600 LTC residents in the current study. Sample size was computed for our primary objective, to detect any differences between our intervention and control trial arms in the incidence of a first delirium episode after 18 weeks of follow-up. We used the Freedman log-rank test method to compare two survivor functions at the end of study follow-up. In a post-hoc secondary analysis of the precursor LTC delirium study, for which we applied the PREPARED Trial inclusion and exclusion criteria [33], we obtained incidence rates that we assume will be experienced by our control group, namely 3.75 cases per 100 person-weeks. We tested different scenarios concerning the size of our sample after 18 weeks of follow-up from baseline. We also tested our assumptions regarding the magnitude of our intervention effect by allowing the reduction in incidence rates to vary between 30 and 73%, as previous studies (in non-LTC settings) have reported this range in variation [1, 18–22]. Using methods of estimation ‘uncorrected’ for cluster randomization, we found that including 180 residents per trial arm (after assuming a 15% rate of attrition over our 18 week follow-up period to account for deaths, transfers and drop-outs) will enable us to detect a 40% reduction in the incidence of delirium (HR = 0.6) with a power of 80% at the 5% level (assuming 2-tailed test and equal group size).

**Fig. 3:**
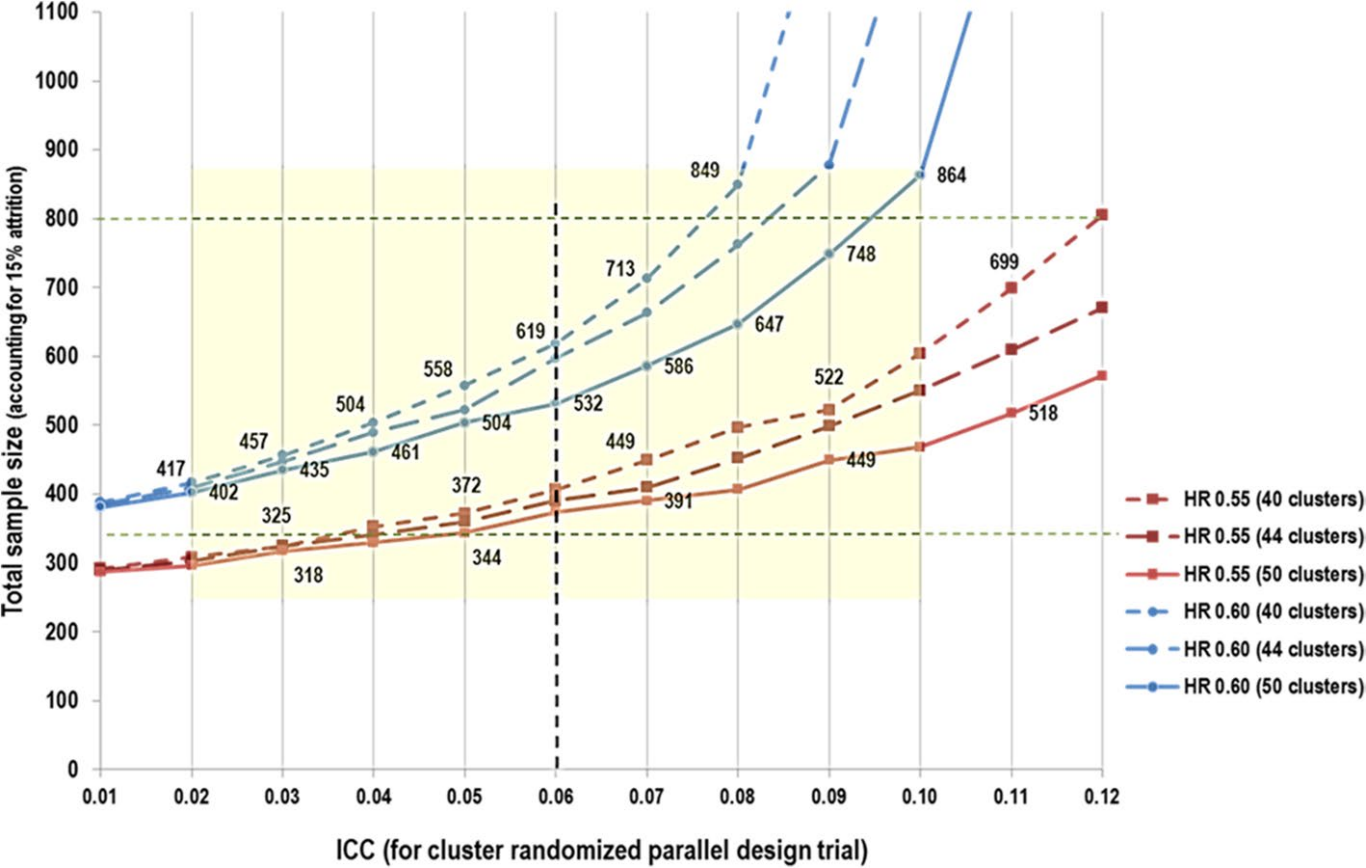
PREPARED Cluster Randomized Trial Sample Size Estimation

The ‘corrected’ sample size for cluster randomization was then computed according to Rutterford et al. (2015) [42], where a design effect was incorporated that inflated the preliminary uncorrected calculation [43]. This design effect depends on two variables: 1) the Intra-cluster Correlation Coefficient (ICC) and 2) the number of clusters, where the total estimated required sample size is directly proportional to the magnitude of ICC and inversely proportional to the total number of clusters participating in the trial. Our sample size analysis, therefore, provides estimates using two conservative values for our expected outcome, and incorporates scenarios for both ICC and the number of clusters (or LTC facilities) that will be included in the study (Fig. 3).

For the purpose of this protocol, we began with an estimated ICC derived from the 2011 Quebec delirium study post-hoc analysis described above. In this dataset, when considering the three facilities that would be representative of the clusters or LTCFs participating in the PREPARED Trial, we found an ICC of 0.06. This estimate should be regarded as quite conservative, given that this ICC was computed using a total of only 3 clusters, whereas our study will be approximately 15 times larger in terms of total cluster size. The number of clusters per arm in our study will vary between 20 (worst case scenario) and 25 (best case scenario). Before performing our CCR procedure, recruited RNs will each be asked to provide the study team with the maximum number of participants that they would be willing to manage as trial participants under the assumption that theirs could be an intervention site. Given that we know a priori that our clusters are likely to be unequal in size, our randomization procedure will use this nurse-to-resident participant ratio as a CCR variable to balance the ratio of intervention-to-control participants in our trial. It should be noted that the range of sample size required varies greatly by ICC: from 864 study subjects within 50 clusters in order to detect a minimum HR of 0.6 in our worst case scenario of an ICC of 0.1, to approximately 300 subjects for HR of − 0.55 and an ICC of 0.02. As such, we plan to enroll about 200–300 residents per intervention arm, for a total enrollment of 400–600 residents, which will account for a liberal 15% estimate for participant attrition. Computations for the above sample size calculations were performed using STATA version 13.0.

### Enrollment and randomization procedure

To ensure a manageable workload for the LTCF nursing staff, the study will enroll only as many residents per team as the participating day shift RN deems to be reasonable. This number will be determined on an individual basis, and will constitute a contract between the research team and the LTCF RN.

Residents who have consented to participate will undergo a two-week delirium prevalence screening using the CAM and the DI (see assessments below) [37–39]. Residents who are delirium-free throughout this two-week period will be randomized for inclusion in the study. The number randomized per unit will be consistent with the number agreed upon by that specific unit nurse.

Residents will be randomized using an electronic data management (EDM) platform provided by the Information Management Services Unit (IMS) at McGill University/Lady Davis Institute of the Jewish General Hospital. IMS will employ simple random sampling, a basic sampling technique where a subset of individuals (a sample) is selected from within a larger set of individuals (a population). In such a method, each individual is chosen entirely by chance, and each member of the population has an equal chance of being included in the sample.

### The PREPARED Trial intervention

This PREPARED Trial intervention was developed by members of our team specifically for the LTC environment using an integrated knowledge translation strategy, which affirmed its feasibility in this setting [27]. The PREPARED Trial intervention is a multicomponent intervention (available in both English and French) consisting of the following:

1. Decision Tree (Fig. 2): This one-page support tool is a 4-step algorithm designed to guide nursing decision-making.
  ***Step 1*** consists of identifying residents at risk of developing delirium using the validated 5-item delirium risk screening tool [36]. For residents deemed at-risk, the tool will then guide the LTC nurse through the three following steps (below).
  ***Step 2*** describes the application of a protocol designed to provide optimal stimulation, and is intended to be implemented by all LTC nursing staff members to all intervention arm participants. The stimulation protocol (STIMUL) involves three actions: 1) surveying the use of eyeglasses and hearing aids, the room lighting, and organization of the space; 2) orienting the resident to time (season, month, day) and to space (city, room); and 3) stimulating the resident with the help of familiar objects, photos, and life histories.
  ***Step 3*** involves a set of specific nursing evaluations to assess the presence of the following five additional modifiable delirium risk factors (i.e., in addition to stimulation): 1) restraint use, 2) prolonged antipsychotic use, 3) dehydration, 4) vision problems, and 5) hearing problems) in preparation for the next step, which represents the specific PREPARED Trial intervention. Evidence of restraint and antipsychotic use are evaluated by examining the resident file, while dehydration is assessed via a thorough inspection of the skin and the mucous membrane of the mouth. Vision problems are evaluated using a tracking test, where residents are asked to follow the trajectory of a red ball within their field of vision. Hearing is assessed via a ‘finger rubbing’ test, where residents are asked to identify or react to sound, depending on their level of cognitive impairment.
  ***Step 4*** describes six specific interventions aimed at reducing the impact of specific risk factors found to be present in Step 3. These six specific interventions are the core of the PREPARED Trial intervention, and are designed to reduce the impact of antipsychotic use, sensory impairment (vision and hearing), restraint use, and dehydration. They are:
    i. Antipsychotic Intervention (ANTIPSY): A letter signed by the study clinician team recommending a re-evaluation of the resident’s need for their antipsychotic medication as per clinical guidelines is inserted into the resident chart so that it may be read and signed by the treating physician.
    ii. Vision Intervention (AVSENS-VISION): A note is made in the resident chart, consultations with an optometrist are suggested, and a relevant intervention based on clinical judgement is suggested and/or implemented as needed.
    iii. Hearing Intervention (AVSENS-AUDIT): A note is made in the resident chart, consultations with an audiologist are suggested, and a relevant intervention based on clinical judgement is suggested and/or implemented as needed.
    iv. Restraint Intervention (CONTEN): All restraints are removed during care that requires the constant presence of a caregiver (nail care, feeding, and dressing changes).
    v. Hydration Intervention (HYDRA): A brightly colored container of fresh water is placed on the bedside table, the resident is offered liquids that s/he enjoys and at least one glass of liquid at each meal, 60-180 ml of liquid is offered during medication administration, and a glass of liquid is offered both after morning care and in the middle of the afternoon.
    vi. Hydration Intervention Plus (HYDRA++): For residents who are hesitant to drink, 60 ml of liquid is offered each time a caregiver enters the room in addition to the HYDRA protocol.

It is important to note that AVSENS-AUDIT represents a novel addition to the PREPARED Trial intervention. AVSENS, which stands for Auditory/Vision/Sensory, expands on the original VISION protocol as published by Voyer et al. (2014) to include both a visual *and* auditory evaluation/intervention [29]. This expansion was created to reflect the fact that hearing impairment (not just visual impairment) has been shown to be a risk factor for developing delirium in this population [21, 44, 45]. Furthermore, existing delirium prevention strategies have been shown to improve clinical outcomes by addressing hearing impairment [21].

In order to account for the possible effects of sensory impairment on cognitive testing [46–49], residents will be offered ‘Pocketalkers’™, or personal amplification devices, to use during trial assessments. Use of these auditory aids has been recommended as one of the strategies to avert the misdiagnosis or over-diagnosis of cognitive impairment in older adults with sensory impairment, and to improve the specificity of cognitive testing [50]. All personal amplification devices will be cleaned and sanitized with alcohol-based sanitation wipes prior to each use.

These six trial interventions will be integrated (where indicated) into the Therapeutic Nursing Plan (TNP) by the LTCF RNs who are mandated to carry out both the antipsychotic and the vision/hearing interventions. The restraint and hydration protocols, however, will be administered by LPNs and PSWs.

2. Instruction Manual: This 3-page, easy-to read, manual provides detailed instructions on how to evaluate the 4 specific delirium risk factors. Specific content of each intervention is explained, and nursing directives are provided.
3. Training Package: Provided by the research staff, the package includes (1): a tailored PowerPoint presentation to nurses (60 min) and LPNs/PSWs (30 min) covering all of the information needed to effectively use the PREPARED Trial intervention; and (2) delirium rounds on the unit during the follow up period (organized by the CRN, as needed) to discuss PREPARED Trial intervention-related issues. Training sessions for regulated staff members (i.e. RNs and LPNs) were accredited by Université Laval.
4. Toolkit**:** This transparent box contains all of the necessary materials to evaluate risk factors (e.g., a red ball for the visual assessment protocol) and implement the interventions (e.g. voice amplifiers, magnifiers), as well as memory aid tools (poster, lanyard memory card, leaflet and notice board listing targeted residents). A box will be provided to each unit in the intervention arm of the study.

The intervention components are based on current best practices in LTC, and are not resource intensive – an important consideration in the context of budgetary constraints and reduced direct care time in this healthcare setting. This trial intervention has been shown to be both feasible and acceptable by Quebec LTC staff [29]. The intervention protocol is low-risk, non-invasive, and personalized to meet the risk profiles of each participant. Participants who no longer meet study inclusion criteria or who are no longer able to engage in the study and its protocols due to disease deterioration (as declared by the primary RN) will stop receiving the intervention and all study evaluations. Trial participants are free to withdraw from the study at any time.

### Adherence to the protocol and nursing staff satisfaction assessment

Adherence to the PREPARED Trial intervention (as documented by the addition of the intervention protocols to the TNP) in the intervention arm will be evaluated by the CRN. In addition, RAs will record the use of hearing/vision aids, and the presence of water and restraints (AVSENS-VISION, AVSENS-AUDIT, HYDRA, HYDRA++, and CONTEN) at every weekly face-to-face assessment with residents during the follow-up period. Nursing staff perceptions of the PREPARED Trial multicomponent intervention will be evaluated at study end using a questionnaire to assess intervention relevance, clarity, perceived burden, and sustainability [29].

### Blinding

LTCFs will not be blinded to group allocation (i.e. control/intervention), as trial arm allocation will obvious (the nursing staff in the intervention group will be trained prior to their participation and receive toolkits and reminder posters, while those in the control group will not). Similarly, the RAs tasked with conducting resident assessments will also not be blinded to group allocation, as they will be constantly exposed to the everyday workings of participating LTCFs. For example, certain visual cues (i.e. materials in the charting room) may indicate the trial status of many LTCFs. However, all research staff members responsible for abstracting information pertaining to modifiable delirium risk factors, medical consultations, and any LTCF transfers will be blinded to resident assessment scores, and should engage in such tasks with impartiality. The research team member administering the CAM/DI for a given resident will also be blinded to the cognitive level assessment of that resident. Finally, all researchers tasked with analyzing the data obtained from this study will be blinded to group allocation, as all LTCF names and randomization statuses will be automatically anonymized prior to data analysis by IMS.

### Assessments

**Fig. 4:**
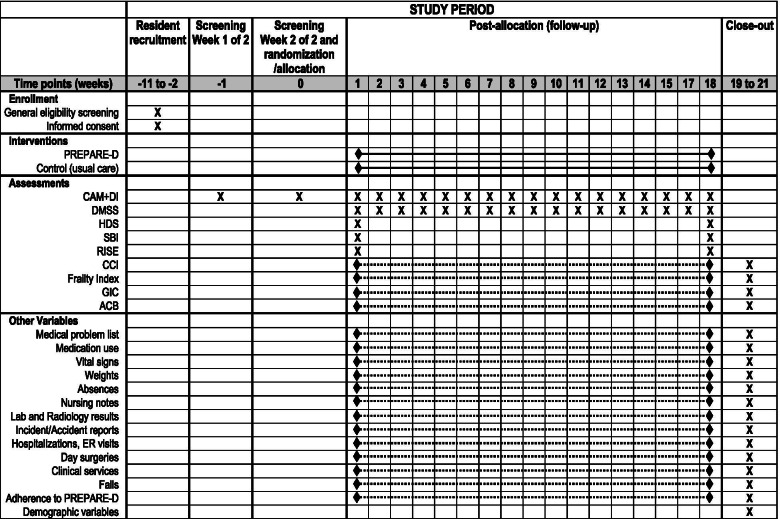
PREPARED Trial SPIRIT Diagram

As part of the eligibility procedure, delirium risk will be assessed using a validated five-item delirium risk screening tool (see “Is the resident at risk?” section of the Decision Tree, Fig. 2) [36]. To be included for study, a resident will require a score of 1 or higher on this scale. Prevalence of delirium will be then assessed weekly for 2 weeks at baseline (screening), and only residents with two consecutive negative CAM scores will be considered for trial inclusion. Delirium incidence and severity (primary outcomes) will be assessed weekly during the 18-week follow-up period. These delirium assessments will be completed using the CAM [37, 38] and the DI [39] as part of a standardized interaction with the resident based on a series of questions adapted using items from various cognitive assessment tools, specifically the Mini-COG (memory) [51–53], Montreal Cognitive Assessment (orientation) and Hierarchical Dementia Scale (concentration) [36, 40, 51, 54, 55]. Functional autonomy will be evaluated at the beginning and end of the follow-up period (weeks 1 and 18) using the Shah version of the Barthel Index [56], an ordinal scale used to measure independence in the activities of daily living (ADLs) adapted to the LTCF environment. Cognitive function will be also be evaluated at these time points using the Hierarchic Dementia Scale (HDS) [55]. Resident comorbidity will be assessed via chart review and reported using the Charlson Comorbidity Index [57] and Geriatric Comorbidity Index [58]. Resident frailty will be assessed via a combination of comorbidity and other measures of well-being, such as functional autonomy, social engagement, and healthcare utilization [59]. Medication information and anticholinergic drug use will also be extracted from resident medical charts, and assessed using the Anticholinergic Burden Scale [60]. Fig. 4 presents a visual representation of the enrolment, intervention, and assessment schedule (SPIRIT Diagram).

### Statistical data analysis

All statistical analyses will be conducted using SAS version 9.4, STATA version 13.0, and R software. Our study has four primary objectives, four secondary, and six tertiary objectives, all aimed at comparing the effect of our PREPARED Trial intervention to that of usual care. Data analysis will be conducted and reported in accordance with current CONSORT guidelines [61, 62].

For each outcome, we will report the estimates of the regression coefficients obtained from the model for the intervention: Hazard Ratio for survival analysis, beta estimate for the continuous outcome, Incident Rate Ratio for the count outcome, and Odds Ratio for the binary outcome. We will also report the ICC (ratio of the variance explained by the LTCF to the total variance explained by the model). All analyses will be conducted at alpha 0.05 and 95% confidence intervals will be reported.

Missing or incomplete outcome data will inevitably appear in our study. Our general approach will be based on intention-to-treat, using inverse probability weighting to account for missing data [63, 64]. To assess the appropriateness of this approach to missing data, we will conduct two sensitivity analyses using the following approaches: 1) inclusion of subjects with complete data only; and 2) use of multiple imputation for missing data [65, 66].

### Quality assurance

In order to ensure that assessments are standardized across evaluators, RAs and CRNs were trained and supervised in Quebec City by a world-renown geriatric nurse with prior experience administering the PREPARED Trial intervention (P. Voyer, PhD). These training sessions included bedside practice. In addition, to mitigate the risk of missing data due to illness or personnel attrition (which is a real possibility given the continuous and inflexible nature of the assessment schedule), several additional researchers will also be trained in study procedures as replacement evaluators, and the study team will be coached in how to train new employees (i.e. ‘train-the-trainer’). Finally, the research staff will also be provided with “booster sessions” periodically, in order to ensure consistency in the administration of the assessments.

The CRN was trained and coached by the geriatric nurse. In turn, the CRN will offer coaching sessions throughout the study to all participating nurses. This will help to ensure standardized implementation of the PREPARED Trial intervention across nursing units.

We will also conduct inter-rater reliability testing during the two-week baseline assessments (prevalence screening), in order to ensure standardized training and assessments [33]. During this period, each RA will accompany a fellow RA on three delirium assessments, so as to independently observe and score the same resident under the same conditions. RAs will also conduct the weekly follow-up assessments in rotating shifts (i.e. will spend no more than three consecutive weeks with the same participants), so as to ensure that residents are assessed by multiple RAs and that assessment results are not nested within an individual assessor.

### Data monitoring and management

The logistical demands associated with large randomized trials involving intensive assessment schedules can be overwhelming. As such, we have contracted with the IMS Unit at McGill University/ the Lady Davis Institute of the Jewish General Hospital to provide an EDM platform that is accessible through a secured online portal. Research staff will enter all assessment scores, including all baseline, weekly, and end of follow-up scores, directly into personal computers (in tablet form) at the point of data collection. The same holds true for all data abstracted from resident charts. All tablets will have access to the Internet, and data collection modules will include scheduling functions for clinical assessments based on the week of follow-up for each study resident. The PREPARED Trial Coordinator and the Principal Investigator will oversee all management of the EDM platform, in collaboration with the IMS team. Basic information (i.e. name, floor, and contact information) pertaining to residents and nursing staff will be entered into the EDM platform. The Clinical Trial Coordinator will also monitor participant retention and follow-up on a weekly basis using the EDM platform. All information collected prior to participant withdrawal will be kept, analyzed or used to ensure the integrity of the project.

All sensitive personal information pertaining to residents and staff members that is not needed for analysis (but that will facilitate data collection, such as name and resident date of birth) will be stored separately and encrypted in a non-searchable table to preserve confidentiality. As a result of this feature, all data that are extracted from the database by the researchers will be automatically anonymized prior to data analysis. Study data will be stored on a secured online portal by IMS, until such time as it is extracted and saved by the Principal Investigator (who will manage the final trial dataset). Given that our trial intervention involves recognized good clinical practices, establishing a Data Safety and Monitoring Board was deemed to be unwarranted.

## Discussion

This study will both advance health-related knowledge and significantly improve health outcomes via the demonstration of the effectiveness of the PREPARED Trial intervention in preventing delirium incidence, severity, frequency and duration of delirium episodes among at-risk LTC residents. New evidence generated by this trial will contribute significantly to the development of clinical guidelines for delirium prevention in this frail elderly population, and will provide a blueprint of a program that can be transferred to other LTCFs around the world. Due to the high prevalence of delirium in this population and its serious consequences on morbidity and mortality, this thorough and well-designed large-scale intervention has the potential to reduce healthcare costs [67] and to significantly improve the quality of LTC in Canada and beyond.

## Data Availability

A de-identified dataset may be made available upon reasonable request of the corresponding author once the study is complete.

## Abbreviations

ACB: Anticholinergic Burden Scale
ANTIPSY: Antipsychotic intervention component of the PREPARED Trial
AVSENS-AUDIT: Hearing intervention component of the PREPARED Trial
AVSENS-VISION: Vision intervention component of the PREPARED Trial
CAM: Confusion Assessment Method
CCI: Charlson Comorbidity Index
CCR: Covariate constrained randomization
CONTEN: Restraint intervention component of the PREPARED Trial
CRN: Clinical research nurse
DI: Delirium Index
DMSS: Delirium Motor Subtype Scale
EDM: Electronic data management
GIC: Geriatric Index of Comorbidity
HDS: Hierarchic Dementia Scale
HELP: Hospital Elder Life Program
HYDRA: Hydration intervention component of the PREPARED Trial
HYDRA++: Hydration plus intervention component of the PREPARED Trial
ICC: Intra-cluster Correlation Coefficient
IMS: Information Management Services, the unit at McGill University/ the Lady Davis Institute of the Jewish General Hospital that will provide the electronic data management platform
LPN: Licensed Practical Nurse
LTC: Long-term care (the study setting)
LTCF: Long-term care facility
MDPP: Multicomponent delirium prevention program
PREPARED: PREvention Program for Alzheimer’s RElated Delirium
PSW: Personal Support Worker
RA: Research Assistant
RISE: Resident Index of Social Engagement
RN: Registered Nurse (or LTCF nurse at a long-term care site)
SBI: Shah version of the Barthel Index
STIMUL: Stimulation component of the PREPARED Trial intervention
TCPS: TriCouncil Policy Statement
TNP: Therapeutic Nursing Plan
